# Evidence of Spondyloarthropathy in the Spine of a Phytosaur (Reptilia: Archosauriformes) from the Late Triassic of Halberstadt, Germany

**DOI:** 10.1371/journal.pone.0085511

**Published:** 2014-01-15

**Authors:** Florian Witzmann, Daniela Schwarz-Wings, Oliver Hampe, Guido Fritsch, Patrick Asbach

**Affiliations:** 1 Museum für Naturkunde, Leibniz-Institut für Evolutions- und Biodiversitätsforschung, Berlin, Germany; 2 Leibniz-Institut für Zoo- und Wildtierforschung (IZW) im Forschungsverbund Berlin e.V., Berlin, Germany; 3 Institut für Radiologie, Charité - Universitätsmedizin Berlin, Berlin, Germany; University of Birmingham, United Kingdom

## Abstract

Pathologies in the skeleton of phytosaurs, extinct archosauriform reptiles restricted to the Late Triassic, have only been rarely described. The only known postcranial pathologies of a phytosaur are two pairs of fused vertebrae of “*Angistorhinopsis ruetimeyeri*” from Halberstadt, Germany, as initially described by the paleontologist Friedrich von Huene. These pathologic vertebrae are redescribed in more detail in this study in the light of modern paleopathologic methods. Four different pathologic observations can be made in the vertebral column of this individual: 1) fusion of two thoracic vertebral bodies by new bone formation within the synovial membrane and articular capsule of the intervertebral joint; 2) fusion and conspicuous antero-posterior shortening of last presacral and first sacral vertebral bodies; 3) destruction and erosion of the anterior articular surface of the last presacral vertebra; and 4) a smooth depression on the ventral surface of the fused last presacral and first sacral vertebral bodies. Observations 1–3 can most plausibly and parsimoniously be attributed to one disease: spondyloarthropathy, an aseptic inflammatory process in which affected vertebrae show typical types of reactive new bone formation and erosion of subchondral bone. The kind of vertebral shortening present in the fused lumbosacral vertebrae suggests that the phytosaur acquired this disease in its early life. Observation 4, the smooth ventral depression in the fused lumbosacral vertebrae, is most probably not connected to the spondyloarthropathy, and can be regarded as a separate abnormality. It remains of uncertain origin, but may be the result of pressure, perhaps caused by a benign mass such as an aneurysm or cyst of unknown type. Reports of spondyloarthropathy in Paleozoic and Mesozoic reptiles are still exceptional, and our report of spondyloarthropathy in fossil material from Halberstadt is the first unequivocal occurrence of this disease in a Triassic tetrapod and in a phytosaur.

## Introduction

**Figure 1:**
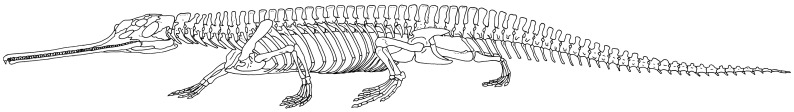
Reconstruction of a phytosaur skeleton (*Rutiodon*), redrawn from [55]. Phytosaurs superficially resemble crocodiles in their habitus and probably had a similar amphibious lifestyle and ecology.

The Phytosauria Jaeger, 1828 (also known as Parasuchia Huxley, 1875) comprise a clade of extinct reptiles known exclusively from Upper Triassic sediments. Phytosaurs are among the most common tetrapods during this time interval with a nearly world-wide distribution [1], [2]. They resemble crocodiles in their habitus (Fig. 1), including the skull shape with an elongate rostrum, the presence of a double-row of dorsal osteoderms covering the trunk and tail, and the long, muscular swimming tail [3]–[11]. Some phytosaurs such as *Mystriosuchus* and *Rutiodon* possessed long, slender and tubular snouts, thus resembling superficially extant gharials; other forms such as *Nicrosaurus* had more massive and deeper rostra that may bear conspicuous dorsal crests and protuberances. Despite all superficial similarities, the skeleton of phytosaurs differs in many respects from that of crocodyliforms, in particular in the position of the external nares close to the orbits and the lack of a secondary palate [2], [8]. Nevertheless, the crocodile-like habitus of phytosaurs is generally regarded as an adaptation to a similar, amphibious lifestyle and ecology, and phytosaurs have even been designated as the “ecological forerunners” of crocodyliforms [1], [8], [12].

Because of their relatively uniform habitus, monophyly of the clade Phytosauria has never been questioned. Traditionally, phytosaurs have been viewed as archosaurs placed rather basally on the crurotarsan line (i.e., archosaurs more closely related to crocodylomorphs than to birds) [13]–[15]. A recent phylogenetic analysis, however, placed Phytosauria in a more basal position as sister taxon to Archosauria [16].

Numerous remains of phytosaurs have been found and described during the past nearly 200 years since the group was first discovered and named (Phytosauridae) by Jaeger [17], but among these specimens are only a few with pathologies which are almost restricted to the snout region. The scarcity of postcranial pathologies might be related to the fact that, whereas phytosaur skulls are relatively common in the fossil record, articulated or at least associated postcranial skeletons are rare. Pathologies such as fracture and infection of the snout have been described in phytosaurs [18]–[21]. Furthermore, the prominent crests and protuberances in the rostral region of phytosaurs such as *Nicrosaurus* or *Angistorhinus* were interpreted as callosities following injury caused by territorial fights [22]. This view, however, was later challenged and these eminences were reinterpreted as not pathologic for two reasons: first, there is no evidence of bite wounds, and second, the protuberances are always symmetrical and arranged in the median line on the rostrum [23].

The only postcranial pathologies known so far in a phytosaur are two pairs of fused vertebrae from the Central European taxon “*Angistorhinopsis ruetimeyeri*” described by Friedrich von Huene [24], who interpreted the fused vertebrae in this specimen as long-term consequences of trauma, possibly fracture, that the animal had experienced earlier in its life. In this study, we redescribe the two pairs of fused vertebrae from von Huene’s specimen in more detail. We provide CT scans of the vertebrae and discuss their pathologies in the light of modern paleopathology, which has undergone great advances both in diagnostic and methodological aspects since the time of von Huene’s work [25].

## Materials and Methods

The pathologic vertebrae investigated in this study are catalogued and stored at the collection of the Museum für Naturkunde Berlin, Germany (institutional abbreviation: MB). They encompass the two specimens MB.R.1972 (fused thoracic vertebrae, [24]: fig. 100a, b) and MB.R.1973 (fused last presacral and first sacral vertebra, [24]: fig. 101a, b). They were found in Upper Triassic sediments (Rhaetian) of the Baereck’sche Tongrube (clay pit) near Halberstadt, central Germany, in an excavation led by Otto Jaekel in 1909–1912 [26]. The quarry yielded a diverse Late Triassic vertebrate fauna including the prosauropod *Plateosaurus*, theropod remains such as *Halticosaurus*, the turtle *Proganochelys*, sauropterygians, the enigmatic diapsid reptile *Elachistosuchus*, and the stereospondyl amphibians *Plagiosaurus* and *Cyclotosaurus* (*Hercynosaurus*) [26]. The phytosaur material collected from Halberstadt includes a smaller and larger forms [24]. The small phytosaur was identified as *Mystriosuchus* sp. [24] and is represented by several disarticulated skeletal remains such as teeth, osteoderms, fragments of skull and lower jaw, vertebrae, ribs, and parts of the limb girdles and extremities. From the large phytosaur, isolated teeth, fragments of the palate, vertebrae, left clavicle and coracoid, parts of the pelvis, femur and metatarsals were found [24]. Von Huene [24] referred this material, together with additional skeletal remains found in the Rhaetian strata of Salzgitter (northern Germany), to *Angistorhinopsis ruetimeyeri* ( = *Mystriosuchus ruetimeyeri* von Huene, 1911), a taxon first described from the Rhaetian sediments of Niederschönthal near Basel, Switzerland. However, the type material of this taxon in the Naturhistorisches Museum Basel (NMB) is most probably not diagnostic (Richard Butler, pers. comm. to DSW 2013), and as a result *Angistorhinopsis ruetimeyeri* cannot be considered to be valid. Pending a redescription of the complete phytosaur material from Halberstadt and Salzgitter, which is in preparation by the present authors, the material of the large form from Halberstadt is currently only referable to Phytosauria indet.

The pathologic vertebrae investigated in this study belong to the large form. The similarities in size, color and preservation of all material attributed to the large form, as well the overall dominance of partly complete and associated reptile skeletons in the quarry [26], support our view that the skeletal parts of the large form belong to a single individual. Comparison of the vertebrae and femoral remains of the large specimen from Halberstadt show that it probably reached a total body length of seven or eight meters, which is huge in comparison with most other phytosaurs [27]. Apart from the large size of the specimen, the fact that the neurocentral sutures are closed in the preserved vertebrae (including those of the anterior thoracic region) indicates an advanced ontogenetic age of the animal [28]. Preservation of the phytosaur material from Halberstadt is throughout good and the specimens are not distorted or compressed. Therefore, it can be ruled out that the anomalies described are caused by taphonomic or preservational effects.

The two pathologic specimens and a medial caudal vertebra (MB.R.4227) of the same individual without evidence of pathologies were scanned using a standard multi-detector computed tomography scanner. In Table 1, vertebrae of the pseudopalatine phytosaurs *Nicrosaurus kapffi* and *Mystriosuchus* sp. from the Late Triassic of southwestern Germany are listed. They are housed at the Staatliches Museum für Naturkunde Stuttgart, Germany (SMNS), and were investigated for comparison with the Halberstadt material.

**Table 1:** List of phytosaur vertebrae studied for comparison with the phytosaur vertebrae from Halberstadt.

Inventory no.	Taxon	Designation	References
SMNS 4384	*Nicrosaurus kapffi*	“lumbar” vertebra	–
SMNS 12671/2	*Nicrosaurus kapffi*	three cervical vertebrae	[55]: fig. 1
SMNS 5999	*Nicrosaurus kapffi*	dorsal vertebra	[24]: fig. 9b
SMNS 12671/3	*Nicrosaurus kapffi*	7^th^ –10^th^ presacral vertebrae	[24]: fig. 3
SMNS 12671/4	*Nicrosaurus kapffi*	seven proximal caudal vertebrae	[24]: fig. 17
SMNS 5719/6	*Nicrosaurus kapffi*	proximal caudal vertebra	[24]: fig. 15a–d
SMNS 5719/7	*Nicrosaurus kapffi*	proximal caudal vertebra	[24]: fig. 16
SMNS 91560	?*Nicrosaurus kapffi*	cervical vertebra	[24]: fig. 4a–c
SMNS 5719/10	?*Nicrosaurus kapffi*	first sacral vertebra	[24]: fig. 10a–d
SMNS 5719/5	?*Nicrosaurus kapffi*	second sacral vertebra	[24]: fig. 11a–c
SMNS 4384/2	*Mystriosuchus* sp.	first sacral vertebra	[24]: fig. 12
SMNS 5719	*Mystriosuchus* sp.	tail vertebra	[24]: fig. 21
SMNS 5719/4	*Mystriosuchus* sp.	proximal caudal vertebra	[24]: fig. 20a, b
SMNS 5719/3	*Mystriosuchus* sp.	proximal caudal vertebra	[24]: fig. 19

Abbreviation: SMNS – Staatliches Museum für Naturkunde Stuttgart, Germany.

The computed tomography was performed in the Leibniz Institute for Zoo- and Wildlife Research (IZW) in Berlin, using a high-resolution Multislice-CT scanner (Aquilion CX). A spiral scan with a 0.5 mm interval was made, leading to DICOM stacks of between 326 and 927 images, according to the size of the objects scanned. The settings were 135 kV and 250 mA. Data were reconstructed in bone algorithm with a VITREA Workstation (Vitrea 2, Version 4.1.2.0, Vital Images, Inc, Minnetonka, Minnesota, USA) and an Oxirix Workstation (Osirix V 3.9.4, 64 Bit). The CT data are deposited in the Museum für Naturkunde Berlin, Germany and can be made available by the present authors for the purpose of scientific study.

## Results

### External Morphology

**Figure 2:**
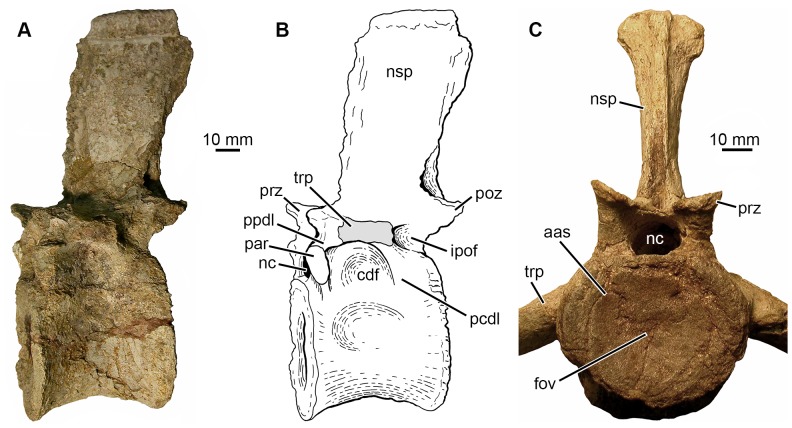
Morphology of “normal” phytosaur vertebrae (*Nicrosaurus kapffi*). **A, B.** SMNS 5999, left lateral view of presacral vertebra. **C.** SMNS 4384, posterior presacral “lumbar” vertebra in anterior view. Abbreviations: aas, anterior articular surface; cdf, centrodiapophyseal fossa; fov, fovea; ipof, infrapostzygapophyseal fossa; nc, neural canal; nsp, neural spine; par, parapophysis; pcdl, posterior centrodiapophyseal lamina; poz, postzygapophysis; ppdl, paradiapophyseal lamina; prz, prezygapophysis; trp, transverse process.

**Figure 3:**
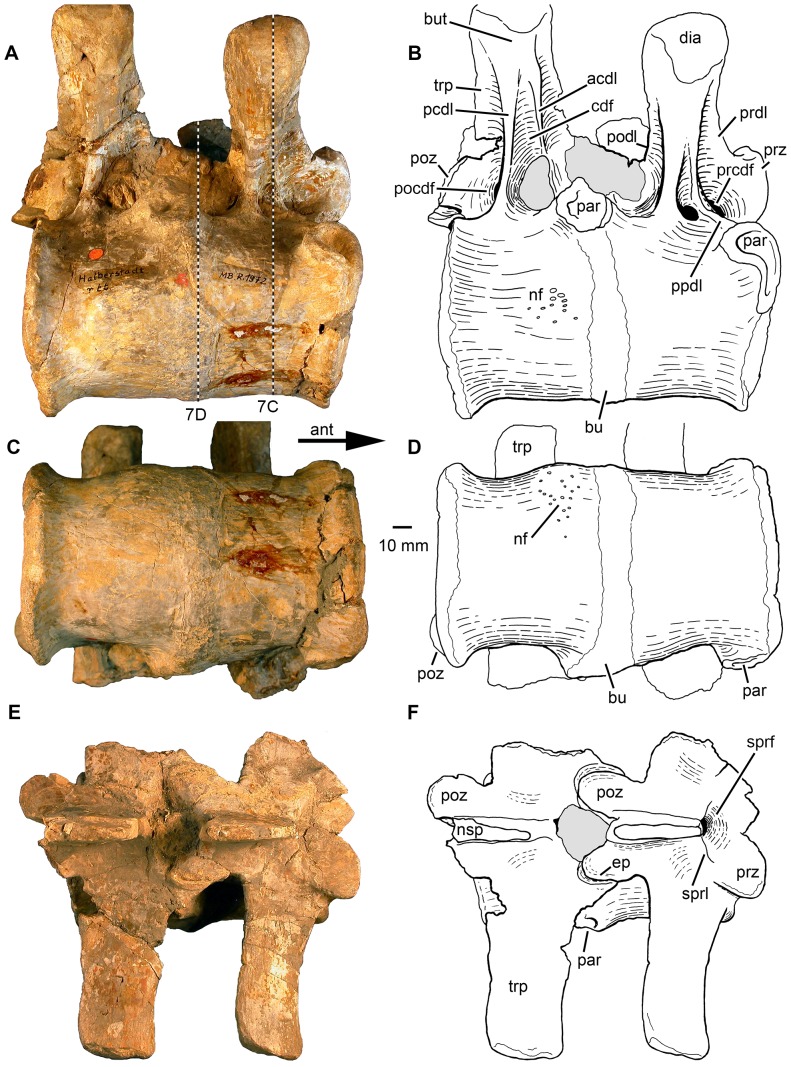
Phytosauria indet. from the Late Triassic of Halberstadt. Fused vertebrae MB.R.1972 derived from the thoracic region. **A, B.** Right lateral view. **C, D.** Ventral view. E, F. Dorsal view. The dashed lines in (A) show the location of sections illustrated in Figure 7C, D. Abbreviations: acdl, anterior centrodiapophyseal lamina; ant, anterior; bu, bulge; but, buttress; cdf, centrodiapophyseal fossa; dia, diapophysis; ep, epipophys; nf, nutrient foramina; nsp, neural spine; par, parapophysis; pcdl, posterior centrodiapophyseal lamina; pocdf, postzygapophyseal centrodiapophyseal fossa; podl, postzygodiapophyseal lamina; poz, postzygapophysis; ppdl, paradiapophyseal lamina; prcdf, prezygapophyseal centrodiapophyseal fossa; prdl, prezygodiapophyseal lamina; prz, prezygapophysis; sprf, spinoprezygapophyseal fossa; sprl, spinoprezygapophyseal lamina; trp, transverse process.

**Figure 4:**
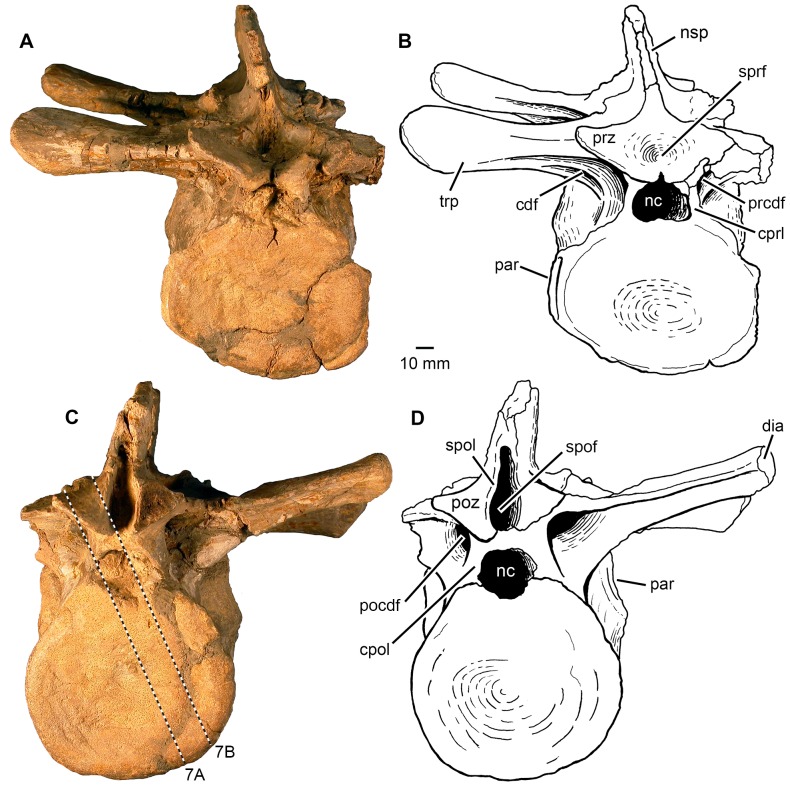
Phytosauria indet. from the Late Triassic of Halberstadt. Fused vertebrae MB.R.1972 derived from the thoracic region. **A, B.** Anterior view. **C, D.** Posterior view. The dashed lines in (C) show the location of sections illustrated in Figure 7A, B. Abbreviations: cdf, centrodiapophyseal fossa; cpol, centropostzygapophyseal lamina; cprl, centroprezygapophyseal lamina; dia, diapophysis; nc, neural canal; nsp, neural spine; par, parapophysis; pocdf, postzygapophyseal centrodiapophyseal fossa; poz, postzygapophysis; prcdf, prezygapophyseal centrodiapophyseal fossa; prz, prezygapophyses; spof, spinopostzygapophyseal fossa; spol, spinopostzygapophyseal laminae; sprf, spinoprezygapophyseal fossa; trp, transverse process.

**Figure 5:**
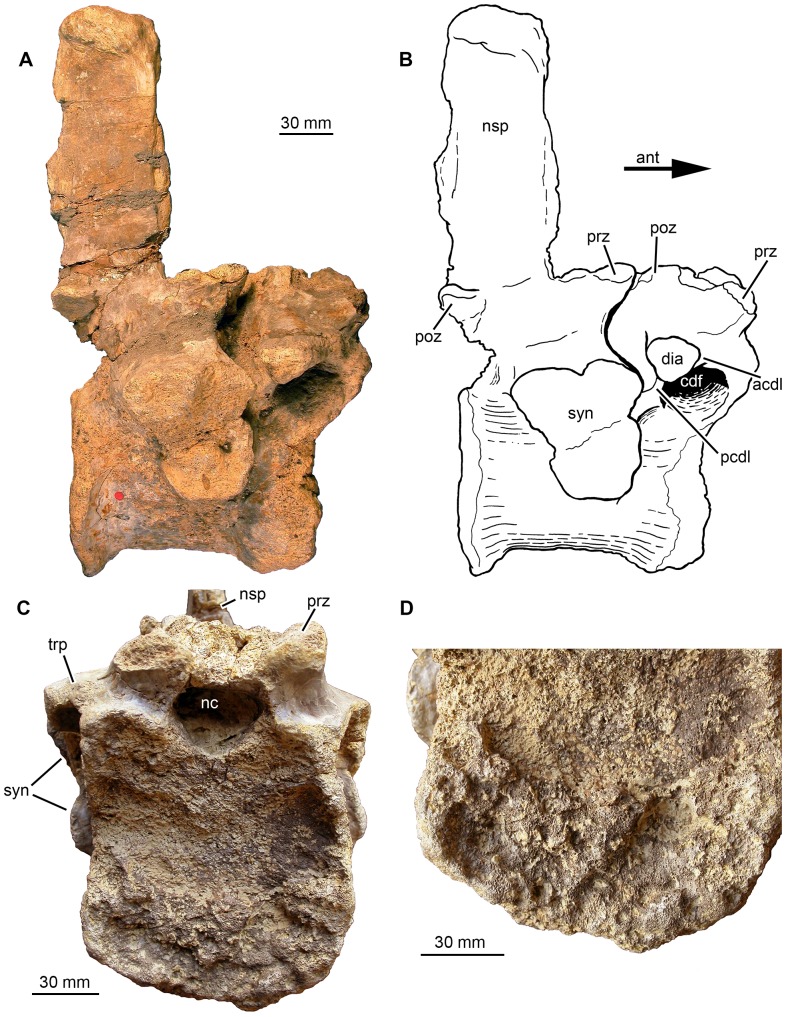
Phytosauria indet. from the Late Triassic of Halberstadt. Fused vertebrae MB.R.1973 derived from the lumbosacral region. **A, B.** Right lateral view. **C.** Anterior endplate showing severe destruction of its surface. **D.** Close up of the ventral part of the anterior endplate. Abbreviations: acdl, anterior centrodiapophyseal lamina; ant, anterior; cdf, centrodiapophyseal fossa; dia, diapophysis; nc, neural canal; nsp, neural spine; pcdl, posterior centrodiapophyseal lamina; poz, postzygapophysis; prz, prezygapophysis; syn, synapophysis; trp, transverse process.

**Figure 6:**
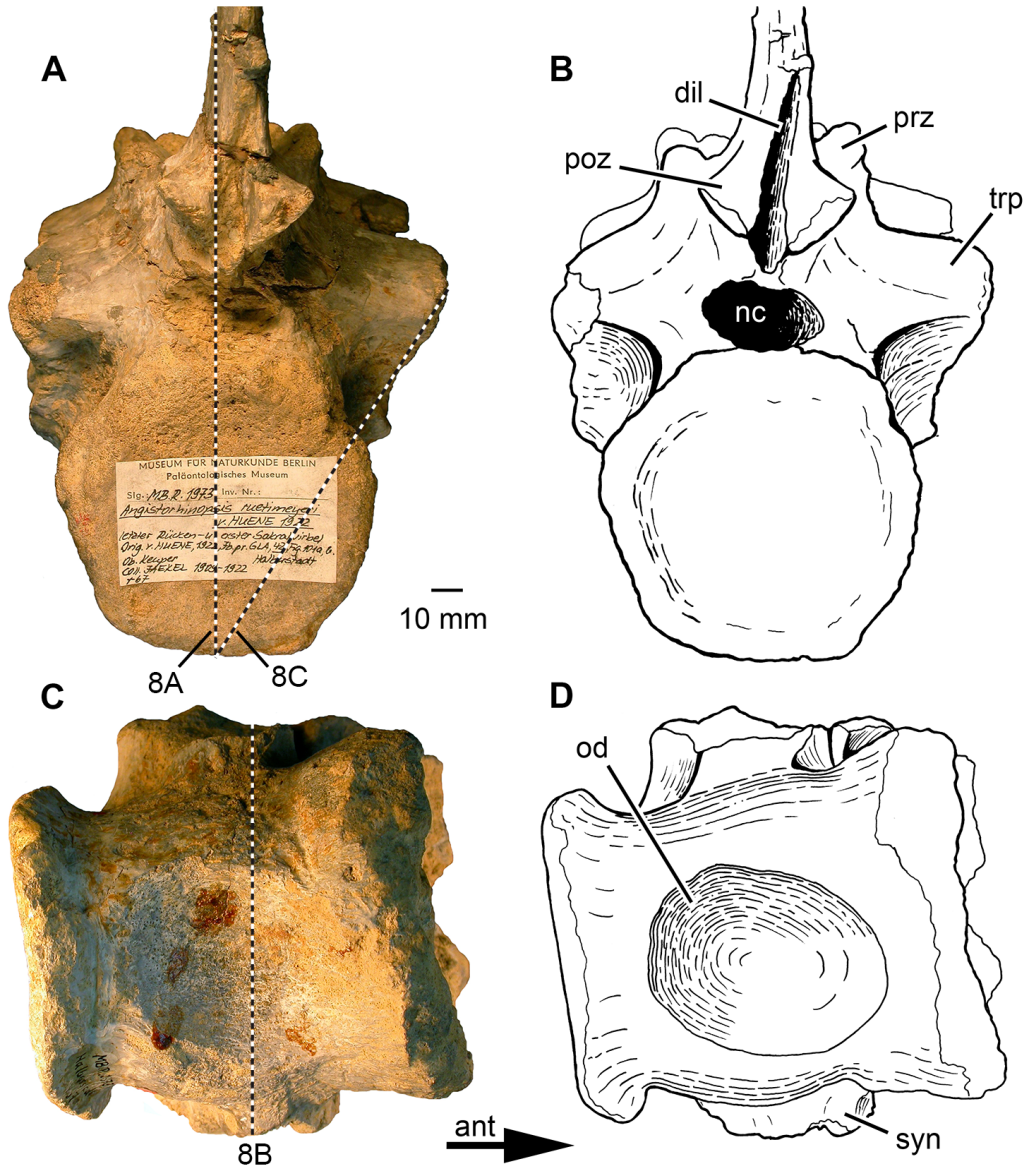
Phytosauria indet. from the Late Triassic of Halberstadt. Fused vertebrae MB.R.1973 derived from the lumbosacral region. **A, B.** Posterior view. **C, D.** Ventral view, showing ovate depression on the ventral surface of the synostosed vertebral bodies. The dashed lines in (A) show the location of sections illustrated in Figure 8A, C; the dashed line in (C) shows the location of section illustrated in Figure 8 B. Abbreviations: ant, anterior; dil, depression for interspinous ligament; nc, neural canal; od, ovate depression; poz, postzygapophysis; prz, prezygapophysis; syn, synapophysis; trp, transverse process.

Figure 2 depicts the morphology of “normal” (i.e. vertebrae not displaying pathologies) presacral vertebrae of a phytosaur (*Nicrosaurus kapffi*) from the Late Triassic of southern Germany for comparison with the pathologic vertebrae from the phytosaur from Halberstadt. The resemblance of the well-developed vertebral fossae and laminae associated with the neural spines, diapophyses and parapophyses of phytosaur vertebrae to those features present in the vertebrae of saurischian dinosaurs was emphasized already in the early literature [3], and saurischian-likevertebral laminae have also been observed in further non-dinosaurian archosaurs [29]–[31]. Therefore, in Figures 2–6, we use the terminology of laminae and fossae that is applied for the vertebrae of saurischians [32], [33]. This terminology is used here only in a strict descriptive sense and does not imply homology or the presence of vertebral pneumaticity in the described specimens.

#### Fused vertebrae MB.R.1972

The specimen consists of two fused vertebrae (Figs. 3–4), which were determined by von Huene to be probably the 9^th^ and 10^th^ presacral vertebrae [24]. The position of the parapophyses and their distinct change in outline indicate that both come from the most anterior thoracic region of the body (e.g. [3], [4]), so that the original identification of von Huene [24] is plausible. The two vertebral bodies are fused completely to one another without any superficial trace of an intervertebral suture (Fig. 3). The fusion zone surrounds the vertebral bodies ventrally and thereby extends between the left and the right parapophyses of the 10^th^ presacral vertebra. The fusion zone is slightly elevated and forms a rounded bulge at the vertebral surface that is most strongly developed on the left lateral side. The bone surface of the bulging fusion zone is covered by an irregularly rugose and apparently porous texture, which is most strongly pronounced on the ventral vertebral surface. On the right ventrolateral side, the surface of the 10^th^ vertebral body posterior to the bulge is distinctly swollen. This inflated surface bears a large number of mostly small nutrient foramina (Fig. 3A–D).

The anteroposterior length of both vertebral bodies together (measured on the ventral surface) is 126 mm. Taking the midpoint of the ventral bulge between the bodies as the boundary between the two elements, the 9^th^ vertebral body has a length of 60 mm and the 10^th^ vertebral body has a length of 63 mm. The exposed anterior and posterior articular surfaces of the vertebral bodies are slightly concave (amphicoelous) (Fig. 4). The anterior articular surface of the 9^th^ vertebra is weakly transversely elliptic in outline and measures 81 mm in width and 70 mm in height. The concavity of the articular surface is developed in the medial two-thirds of the area and surrounded by a flat area that is slightly tilted posteriorly especially in its ventral part. In comparison, the posterior articular surface of the 10^th^ vertebra is nearly circular in outline and measures 83 mm in width and 76 mm in height. The concavity of this articular surface occupies approximately four-fifths of the whole articular surface and is surrounded by a flat and slightly anteriorly tilted rim. A circular fovea is located dorsomedially within the concavity. In ventral aspect, the anterior and posterior articular surfaces are transversely expanded relative to the remainder of the ventral surface (Fig. 3C, D). The ventral surface of the bodies is, with exception of the inflated area on the 10^th^ presacral, planar and only slightly concave in lateral view (Fig. 3A, B) rather than being constricted or having an “hourglass form” as described usually for vertebral bodies of phytosaurs (e.g. [3], [24], [28], [34]). Both vertebrae possess well-developed, laterally facing parapophyses (Fig. 3A).

The neural arches of MB.R.1972 are not involved in vertebral fusion, and no abnormal swellings or surface textures can be observed (Fig. 3E, F). The bone surfaces in the region of the neurocentral suture are abraded on the left side of the vertebral bodies, but well preserved on the right side, where it exposes no evidence of a neurocentral suture. The neural arches therefore seem to be completely fused to the vertebral bodies. The massive transverse processes and diapophyses are well preserved on the right side in both vertebrae, whereas those on the left side are broken away.

The transverse process of the 10^th^ presacral vertebra is positioned slightly dorsal to the 9^th^, and its dorsal surface is not tilted anteroventrally, but is instead oriented horizontally (see also [24]). The pre- and postzygapophyses of both vertebrae possess only short peduncles (Fig. 4). On the roof of the neural arch, a well-developed, funnel-shaped spinoprezygapophyseal fossa and a deep spinopostzygapophyseal fossa are present between the pre- and postzygapophyses, respectively. Both fossae merge dorsally into a depression, presumably for the elastic interlaminar ligament. The neural spines are broken in their anterior and dorsal parts, but they can be reconstructed as inclined slightly posteriorly. Both neural spines measure c. 45 mm in length at their base. In dorsal aspect, their apices increase slightly in width in a posterior direction (Fig. 3E, F). The neural canal is transversely broad oval in outline and measures c. 21 mm in width and c.16 mm in height.

#### Fused vertebrae MB.R.1973

This specimen consists of two fused vertebrae (Figs. 5, 6). We follow von Huene [24] in his interpretation of these vertebrae as the posteriormost presacral (posteriormost dorsal) and the anterior sacral vertebra. The huge articulation facet for articulation with the ribs clearly identifies the more posterior vertebra as a sacral vertebra, and the distinctly smaller diapophysis of the anterior vertebra is consistent with the interpretation as the last presacral vertebra. Interestingly, in contrast to most other phytosaurs described, there was no bony fusion of the sacral ribs with at least the first sacral vertebra in this individual.

In contrast to MB.R.1972, the two vertebral bodies of MB.R.1973 seem to have fused into one body without any indication of an intervertebral area between them (Fig. 5A, B). The resulting compound vertebral body is constricted in the middle to resemble an hourglass shape and measures c. 90 mm in length ventrally. However, it has to be taken in account that the anterior articular surface appears to be eroded and has a strongly irregular surface (see below). The anterior articular surface has a rectangular outline with rounded corners and measures 78 mm in width and 88 mm in height. The last presacral vertebra shows severe destruction of its anterior articular surface with exposure of an irregular surface of spongy bone (Fig. 5C, D). Especially deep erosion took place in the middle and dorsal two-thirds of the articular surface, whereas in the ventral third of the articular surface, the surface is irregular and crater-like with projecting portions of spongy bone. The margin of the articular surface forms a rim ventrally and ventrolaterally, but is destroyed laterally at the level of the height of the synapophyses of the posteriorly following first sacral vertebra (Figs. 5A–D). The visible posterior articular surface is smaller and subcircular, with a width of 80 mm and a height of 78 mm. The posterior articular surface is nearly flat (platycoelous), with a weakly concave surface surrounded by a narrow rim that is laterally tilted and rugose (Fig. 6A, B). The lateral surface of the vertebral body and the base of the neural arch bear at mid-length of the body a large and rugose articulation facet for attachment of the first sacral rib. The articulation facet is 52 mm high and divided into a 46 mm long dorsal part and a 33 mm long ventral part, separated by a medial constriction. The dorsal, ventrolaterally directed part belongs to the neural arch of the first sacral vertebra, whereas the ventral part sits medially on the fused vertebral body and faces laterally. Whereas the ventral part of the synapophysis often extends ventrally as far as the base of the vertebral body in phytosaurs, e.g. as in *Machaeroprosopus* [4] and *Nicrosaurus* (SMNS 5719/5), the synapophysis extends only to the ventral third of the body in MB.R.1973 (Fig. 5A, B). This proportional difference might be ascribed to the extraordinary size of the specimen under study as compared to most other phytosaur taxa. On the left lateral side of the posterior edge of the body is a roughened expansion with a concave surface, which is not preserved on the right side. It might have contributed to the parapophysis of the second (not preserved) sacral vertebra and formed its anterior portion. The anterior body shows no remnants of a parapophysis, possibly because of erosion (see above); the parapophysis of the posterior presacral vertebrae in phytosaurs is small and located at the anterodorsal edge of the vertebral body [3]. A conspicuous bowl shaped depression occupies nearly all of the ventral surface of the united fused vertebral bodies (Fig. 6C, D; see also [24]). This depression is deeper on the left than on the right lateral side. It produces a broad bulge on the vertebral body posterolaterally on the left side of the vertebral body. The bone texture within the depression is longitudinally striated.

Similar to MB.R.1972, the neural arches of the two vertebrae of MB.R.1973 are not co-ossified (Fig. 5A, B). However, they deviate from “normal” neural arch morphology in being anteroposteriorly shortened. There is no trace of a neurocentral suture. The transverse process of the anterior neural arch measures 60 mm from the midpoint of the spinoprezygapophyseal fossa to the diapophyseal articular surface. The diapophyseal articular surface is roughly triangular in shape and is short with a length of only 19 mm, which is characteristic for the last presacral vertebra of phytosaurs [3]. The prezygapophyses sit on short peduncles and have well developed subcircular articular areas that are directed at an angle of about 30° to the horizontal plane [24]. The neural spine of the last presacral vertebra is not preserved; according to its remaining base, it was at most 16 mm long and thus rudimentarily developed. The postzygapophyses are not preserved.

The transverse process of the sacral neural arch is massive and measures 65 mm from the midpoint of the spinoprezygapophyseal fossa to the distal end of the process. The prezygapophyses of the first sacral vertebra are much larger than those on the last presacral. As also pointed out by von Huene [24], the postzygapophyses are rather small and do not exceed the length of the vertebral body (Fig. 5A, B). Similarly, Case [5] described the zygapophyses of the sacral vertebrae in a phytosaur from Cerita de la Cruz Creek, Texas as “reduced in size, though still functional”, and comparably small postzygapophyses can be found in sacral vertebrae of *Nicrosaurus kapffi* (SMNS 5719/10). The neural spine of the first sacral vertebra is completely preserved and directed straight dorsally (Fig. 5A, B). The neural spine is 41 mm long at its base and positioned in the posterior half of the sacral neural arch. The process measures 122 mm in height from the base of the postzygapophyses to the apex. The depression for the attachment of the elastic interlaminar ligament [35] is restricted to about the ventral third of the posterior margin of the neural spine. The neural canal is c. 28 mm wide in the posterior vertebra and c. 17 mm high, and therefore is transversely oval in outline.

### Internal Morphology as Revealed by CT-scans

**Figure 7:**
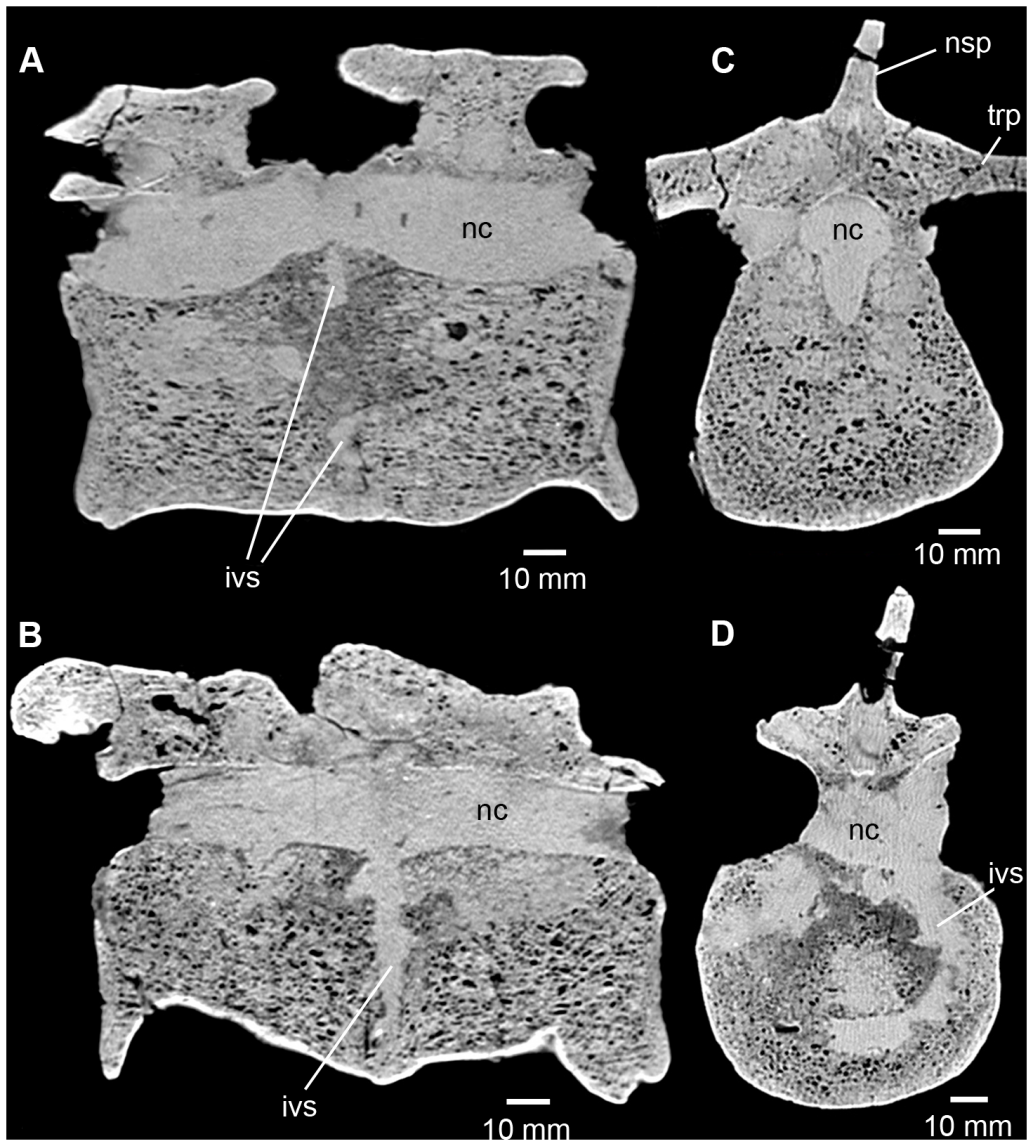
Phytosauria indet. from the Late Triassic of Halberstadt. CT scans of the fused vertebrae MB.R.1972. **A.** Sagittal section along the midline of the vertebrae. **B.** Sagittal section through the left lateral part of the vertebrae. **C.** Transverse section through the anterior vertebra. **D.** Transverse section through the intervertebral space, showing a ring-like structure. For the location of sections, please see Figures 3A and 4C. Abbreviations: ivs, intervertebral space; nc, neural canal; nsp, neural spine; trp, transverse process.

**Figure 8:**
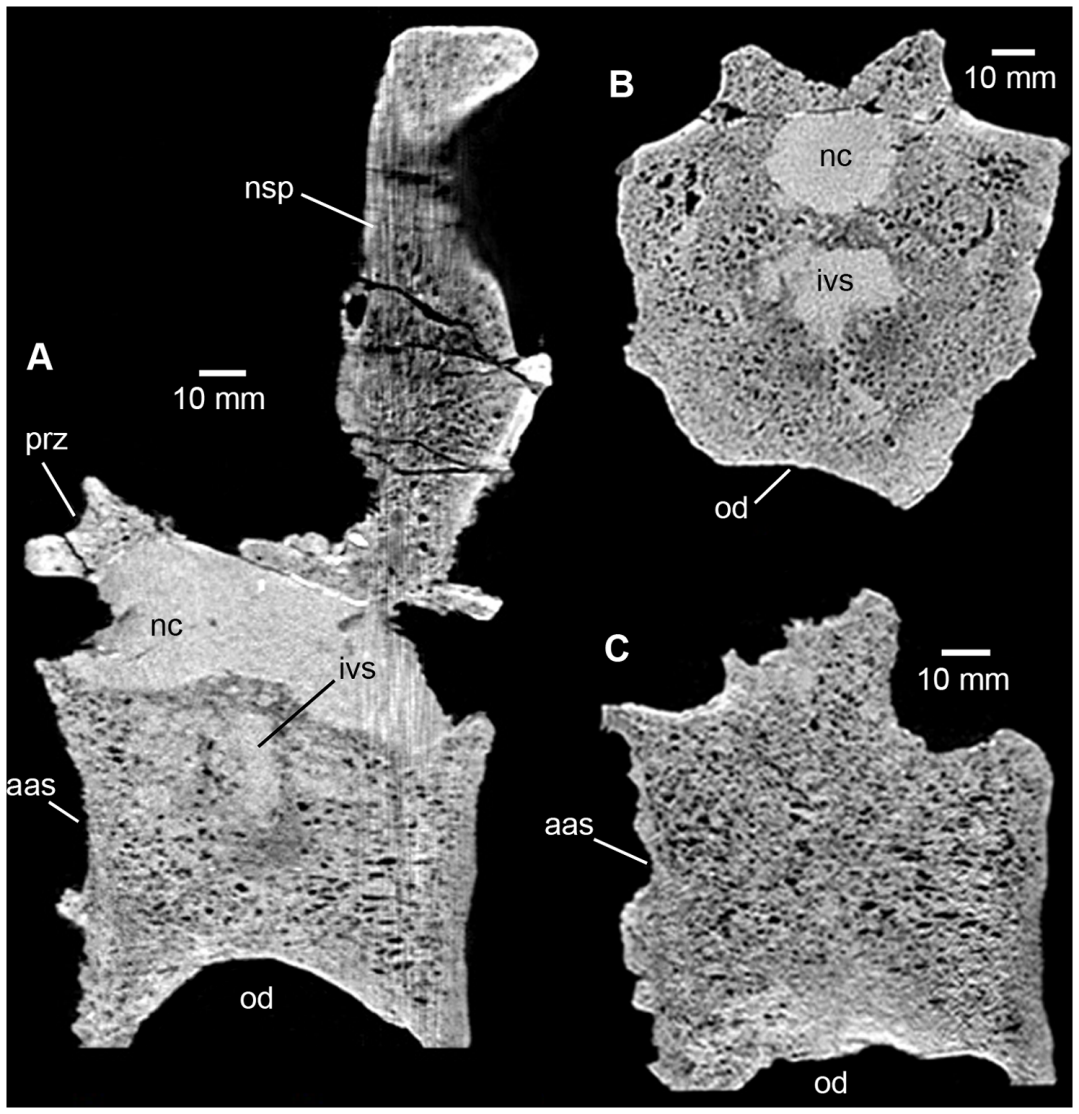
Phytosauria indet. from the Late Triassic of Halberstadt. CT scans of the fused vertebrae MB.R.1973. **A.** Sagittal section along the midline of the vertebrae. **B.** Transverse section through the intervertebral space, showing a simple cavity or lacuna. **C.** Sagittal section through the right lateral part of the vertebrae, showing their complete fusion. For the location of sections, please see Figure 6A, C. Abbreviations: aas, anterior articular surface; ivs, intervertebral space; nc, neural canal; nsp, neural spine; od, ovate depression; prz, prezygapophyses.

The high resolution of the CT-scans has provided a high image quality of the internal structure of the vertebrae and makes it possible to distinguish between cortical and cancellous bone in both specimens (MB.R.1972, 1973). The cortical bone is rather thin and the cancellous bone consists of thin trabeculae both in the vertebral bodies and the neural arches (Figs. 7, 8).

#### Fused vertebrae MB.R.1972

In sagittal section, the sediment-filled neural canal is well visible (Fig. 7A, B). It is constricted at the boundary between the two adjacent vertebrae and expands within each vertebral body in a ventral direction. The largest expansions of the neural canal are not centered within the vertebral bodies, but are positioned in the anterior half of the anterior vertebra and the posterior half of the posterior vertebra, giving the impression that the vertebral bodies are slightly longitudinally compressed in comparison to a non-fused vertebral body. In axial cross section, the neural canal is broadly ovate and the ventral extensions are visible as narrow ventral projections at the anterior and posterior portions of the vertebral bodies (Fig. 7C).

The intervertebral space is well delineated as a ring-like, sediment-filled cavity in axial cross section that occupies the dorsal two-thirds of the cross-sectional area of the vertebral body and dorsally ends directly ventrally to the neural canal (Fig. 7D). The ventral third and the lateral fifths of the vertebral bodies consist purely of undisturbed cancellous bone at the area of the intervertebral space. A circular cavity is located within the ring in the center of the vertebral body and is circumscribed by spongy bone. In sagittal section, the intervertebral space is as equally well discernible as in axial view (Fig. 7A, B).

The internal structure of the vertebral bodies, showing these areas of entirely homogeneous cancellous bone around the intervertebral space, indicates a complete bony fusion of the two vertebral bodies. In contrast, at the anterior articular surface of the anterior vertebra, and the posterior articular surface of the posterior vertebra, thick compact bone is mineralized that forms well defined layers parallel to the articular surface of the vertebra.

#### Fused vertebrae MB.R.1973

In sagittal section, the sediment-filled neural canal is well visible as in MB.R.1972 (Fig. 8A). The canal is medially constricted, denoting the otherwise not clearly visible transition between the two vertebral bodies. Similarly to MB.R.1972, the neural canal of MB.R.1972 expands within each vertebral body in a ventral direction. However, in MB.R.1972 the ventral expansion forms a long concavity in lateral view, which occupies two-thirds of the length of the vertebral bodies. With the neural canal being used to identify and measure the separate vertebral bodies, the vertebral bodies appear to be rather shortened anteroposteriorly in comparison to the “normal” condition. In axial cross section, the neural canal is broadly ovate as in MB.R.1972, with the ventral extensions visible as narrow ventral projections into the vertebral bodies.

A sediment-filled, reduced intervertebral space is visible in the dorsal half of the zone of vertebral fusion, level with the constriction of the neural canal (Fig. 8A). In the ventral half of the vertebral body, only homogenous cancellous bone is present. The intervertebral space is smaller than that of MB.R.1972 in axial view (Fig. 8B). It appears not to be developed in a ring-like structure, but rather seems to represent a simple cavity or lacuna. The transverse section of the CT scans exposes undisturbed, homogenous trabecular morphology in this area, demonstrating that fusion between the vertebral bodies is complete.

The large bowl shaped depression on the ventral surface of the fused vertebral bodies is internally accompanied by compact bone that only minimally differs in thickness and structure from the cortex of the lateral parts of the vertebral body. The above described destruction of the anterior articular surface is well discernible, with the bony projections in the ventral third of the articular surface showing areas of bony sclerosis (Fig. 8A, C).

## Discussion

### Intervertebral Discs or Joints in Phytosaurs?

For a classification of the pathologies present in the phytosaur individual from Halberstadt, it is important to have an idea of the nature of the intervertebral articulation in phytosaurs. Because phytosaurs are extinct reptiles, it is only possible to reconstruct the intervertebral space by direct comparison to extant, analogous and phylogenetically closely related taxa. Phytosaurs can be regarded as archosauromorphs and are deeply nested within reptiles [2], [14], [16]. Extant reptiles therefore provide a good analog for such a reconstruction, in particular extant archosaurs such as crocodylians and birds. Crocodyliforms are a particularly good analog for the phytosaur intervertebral articulation, because they include extant members with generally procoelous vertebrae, but also fossil members with amphi- or platycoelous vertebra, similar to phytosaurs. As recent studies have shown [36], extant reptiles do generally not possess intervertebral discs with an anulus fibrosus surrounding a gelatinous nucleus pulposus, which seems to be restricted to mammals (e.g. [37]). There is evidence that this applies also to dinosaurs, at least hadrosaurs [38].

**Figure 9:**
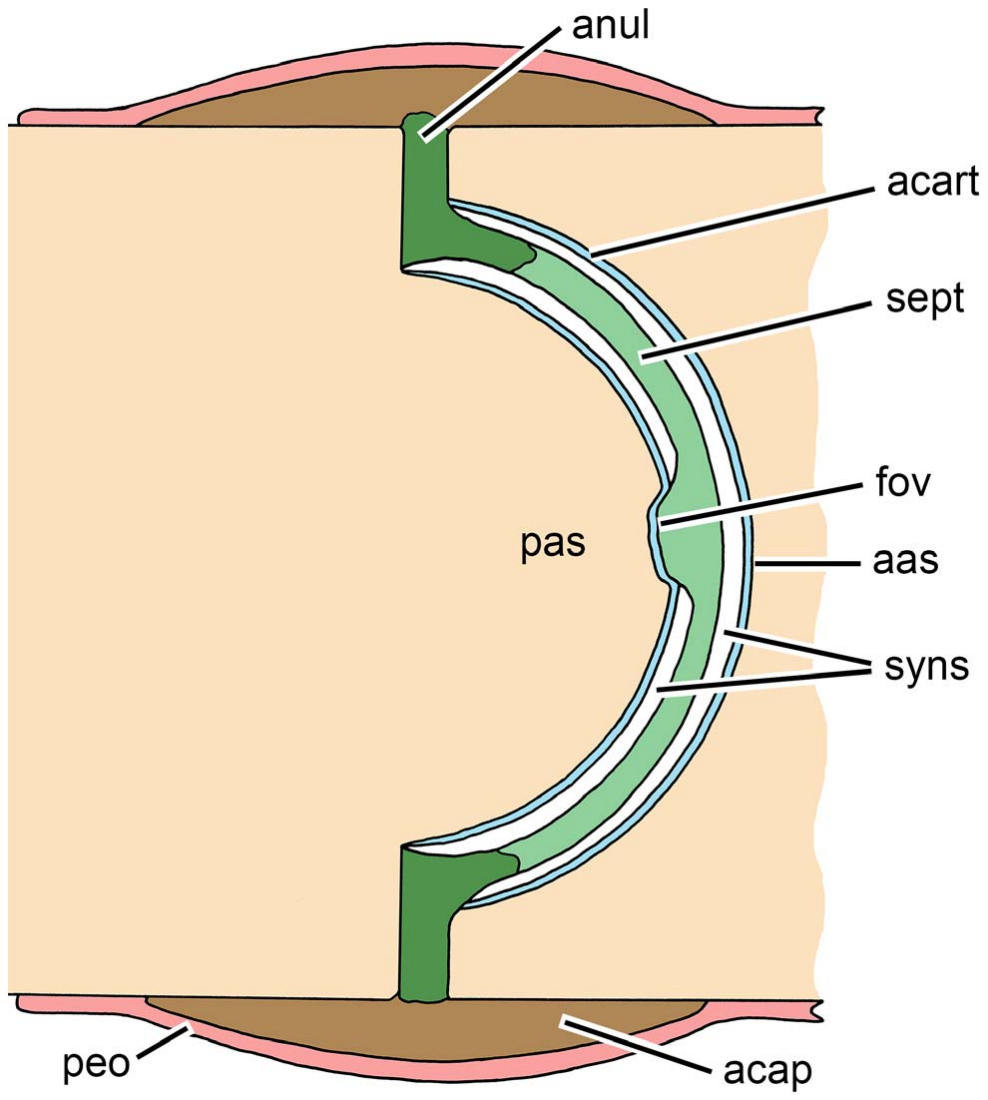
Schematic drawing of the synovial articulation between procoelous thoracic vertebral bodies of an extant crocodylian. Redrawn after [40]; articular cartilage covering the vertebral articular surfaces is added, based on [56]. Abbreviations: aas, anterior articular surface (vertebral fossa); acap, articular capsule; acart, articular cartilage; anul, ring of fibrous cartilage; fov, fovea; pas, posterior articular surface (vertebral condyle); peo, periost; sept, intercorporal septum; syns, synovial space.

In extant birds, which possess heterocoelous intervertebral articulations [36], [39], articulation between cervical vertebral bodies is formed by synovial joints, whereas most thoracic vertebral bodies are separated by intervertebral discs. These discs are solely composed of fibrous cartilage comparable to the mammalian anulus fibrosus, but without a nucleous pulposus [36]. The procoelous vertebral bodies of extant crocodylians are connected by synovial joints, consisting of an outer articular capsule and an inner cavity (synovial space) containing synovial fluid (Fig. 9). The articular capsule, which corresponds to the anulus fibrosus, is made of intersecting collagen fibers that insert at the rugose surface of the ends of the vertebral bodies (rugositas annularis *sensu* [40]). A ring of fibrous cartilage is attached to the roughened periphery of the anterior and posterior faces of the vertebral body and to the inner surface of the capsule. The synovial cavity is divided by a thin intercorporal septum of fibrous cartilage, whose peripheral parts are continuous with the ring of fibrous cartilage, and which is attached to a depression or fovea in the middle of the vertebral condyle of each vertebra [40]. The ring of fibrous cartilage plus intercorporal septum of crocodylians is not a true intervertebral disc, which would connect two adjacent bodies and not contain an articular space [40]. In fossil crocodyliforms with amphicoelous vertebrae, Salisbury & Frey [40] found a rugositas annularis in the peripheral parts of the articular face of the vertebral bodies, but a smooth surface of the vertebral concavities, and concluded that the intervertebral articulation was synovial. The presence of a mammal-like intervertebral disc structure is ruled out for fossil crocodyliforms, because the articular surfaces (or endplates) of mammals are generally roughened due to the attachment of the anulus fibrosus to the anular epiphyes [40].

Examination of the vertebral bodies from the Halberstadt phytosaur and comparative material (vertebral bodies from the presacral and sacral region and the tail of *Nicrosaurus* and *Mystriosuchus* stored at SMNS; Table 1) in the course of the present study revealed the presence of an annular rugosity *sensu* Salisbury & Frey [40] in these specimens. In the best preserved cases, the surface of this structure is composed of striae forming concentric rings (well visible e.g. in SMNS 12671). Longitudinal striae may extend a short distance anteriorly and posteriorly on the lateral and ventral faces of the vertebral bodies, as well visible e.g. in SMNS 10504, and similarly present in dinosaurs such as *Liliensternus* (e.g., MB.R.2175.2.22, 2175.2.27) and *Dysalotosaurus* (e.g., MB.R.1501, 1620, 3551.11). When the bone surface is well preserved, the concave articular surfaces are always smooth. A fovea is present in the center or dorsal to it on both the anterior and posterior articular surfaces (Fig. 2C), although not visible in all specimens, probably because of poor preservation. A similar fovea is developed, for example, on the anterior and posterior articular surfaces of the vertebrae of the theropod dinosaur *Liliensternus* (e.g., MB.R.2175.2.22, 2175.2.27). These osteological correlates indicate the presence of synovial joints with an outer articular capsule, a ring of fibrous cartilage, a spherical synovial cavity and a septum intercorporale in phytosaurs. This reconstruction is also supported by the morphology of extant lepidosaurs, which can have amphicoelous or procoelous vertebral articulations and possess generally synovial joints [41], [42].

### Interpretation of Pathologic Findings

The fused vertebrae in the thoracic plus lumbosacral region including the eroded anterior articular surface in the phytosaur from Halberstadt can most plausibly be explained by spondyloarthropathy, a chronic aseptic inflammatory process, which in humans most often manifests at the sacroiliac joint and the vertebral column, but also involves peripheral (i.e. synovial) joints and tendon insertion sites [25], [43]. Spondyloarthropathy can be considered as a group of different forms of rheumatic diseases, including spondylitis ankylosans (the most frequent type of spondyloarthropathy), psoriatic arthritis, juvenile spondyloarthropathy, reactive arthritis (a reaction to septic inflammation) and others.

In spondyloarthropathy, affected vertebrae show typical changes of reactive new bone formation and erosion. The ventrolateral bulge that is present at the border between the two fused thoracic vertebral bodies (MB.R.1972**)** apparently represents osseous bridging by new bone formation within the synovial membrane and articular capsule of the intervertebral joint. A fracture can be ruled out because no signs of callus formation (i.e. focal irregularity with circumscribed thickening of the cortical bone) are present. Furthermore, the trabeculae of the cancellous bone show no evidence of consolidation that would be expected in case of previous fracture. An acute infection or abscess is unlikely because a lytic component with destruction of the trabecular structure is not present. In addition, there is no evidence of cortical irregularity with periosteal bone deposition which would be seen in case of chronic osteomyelitis.

The fused lumbosacral vertebrae MB.R.1973 do not show the ventrolateral bulge. Instead, they have a waisted appearance and are distinctly shortened. This may be attributed to impaired longitudinal growth of the vertebrae, suggesting that the phytosaur acquired the disease during the growth phase in early life. With their waisted appearance, the fused lumbosacral vertebrae resemble congenital block vertebrae, which are the result of failure of segmentation [44]. However, the length of congenital block vertebrae is not as much reduced as in this specimen [45], whose proportional length is reduced by half compared to “normal vertebrae”; therefore, a congenital block vertebra is rejected here. The reduction in length cannot be attributed to fracture or collapse, because the CT scans reveal homogeneous trabecular structure throughout and no callus formation is present. This applies also to the bowl shaped depression on the ventral surface of the compound vertebral body because CT scans show only a slight bone reaction underlying it. One can only speculate about the nature of this depression. Its smooth nature suggests that among other causes, it may be the result of pressure, perhaps caused by a benign mass (e.g. aneurysm or cyst of unknown type). Similar smooth depressions of the bone surface were recently described in the palate and scapula of Permo-Triassic dicynodonts and interpreted as the result of pressure erosion, caused either by a cyst or hydatid disease, i.e. infection by the cestode *Echinococcus* [46]. However, the cause of the depression in the phytosaur remains speculative but is certainly not connected to spondyloarthropathy. The destroyed anterior articular surface of the last presacral vertebra in MB.R.1973 can best be interpreted as erosion of the subchondral bone surface, which is characteristic of spondyloarthropathy [25]. At this point, the presumed soft-tissue structure between vertebral bodies in phytosaurs comes into play. As discussed above, phytosaurs most probably did not possess a mammal-like intervertebral disc with a nucleus pulposus framed by an anulus fibrosus. Instead, adjacent vertebral bodies were separated by a synovial space, and the articular surfaces of the bodies were covered by cartilage. The disease process must have eroded through the underlying subchondral bone.

Differential diagnoses for spondyloarthropathy are rejected as follows: 1) Infectious arthritis, which may cause a similar destruction of the articular surface and bony bridging between vertebrae [25] is rejected because destruction is not visible in the adjacent vertebra; 2) Spondylitis tuberculosa (or specific spondylitis, i.e. inflammation of one or more vertebral bodies by *Mycobacterium tuberculosis*) is rejected because compression fractures (including kyphosis, i.e. dorsally convex curving of the vertebral column) and lytic areas in the vertebral body are absent; 3) Osteochondrosis intervertebralis (the classic appearance of degenerative disease of the vertebral column) is rejected because of the absence of osteophytes (i.e. bony projections along joint margins) including typical change in the form of the vertebrae; 4) Vertebral tumor is rejected because its destructive alterations are restricted to the vertebral body and not the articular surfaces [47]; 5) Spondylitis ankylosans is rejected because it exhibits pronounced ankylosis (i.e. rigid union) of several vertebral segments [47]; 6) Scheuermann’s disease (i.e. localized osseous defects in the endplate of vertebral bodies) is rejected because of the absence of a) circumscribed defects (Schmorl’s nodes) in the articular surface and b) of wedge-like ventral narrowing (i.e. kyphosis) of the vertebral bodies [47], [48].

### Etiology

Four different pathologic observations can be made in the vertebral column of the phytosaur individual from Halberstadt: 1) fusion of two thoracic vertebral bodies; 2) fusion and shortening of last presacral and first sacral vertebral bodies; 3) erosion of the anterior articular surface of the last presacral vertebra; and 4) smooth depression on the ventral surface of the fused last presacral and first sacral vertebral bodies. Observations 1–3 can most plausibly and parsimoniously be attributed to one disease: spondyloarthropathy. The evidence described above strongly suggests this diagnosis, although the zygapophyseal joints do not show erosion or fusion [49]. The vertebral shortening present in the fused lumbosacral vertebra of the phytosaur indicates that the individual acquired this disease as a juvenile. Although the phytosaur was apparently affected by spondyloarthropathy almost throughout its entire life, the unusual body length of seven to eight meters indicates that the phytosaur was probably several years old and was not severely hampered by the disease.

## Conclusions

Spondyloarthropathy is a well-known aseptic inflammatory disease process in humans which can cause fusion of joints in the axial and appendicular skeleton [25], [50]. The bony bridging of vertebral bodies in humans and other mammals occurs by ossification within the outer layer of the intervertebral disk, the anulus fibrosus. Apart from mammals, spondyloarthropathy has been demonstrated also in extant and extinct reptiles like lizards (including mosasaurs), crocodiles and dinosaurs in recent years, although reports of this disease in Paleozoic and Mesozoic reptiles is still exceptional (e.g. [25], [49], [51]–[53]). Because reptiles lack intervertebral disks but instead possess synovial joints between vertebral bodies, ossification takes place within the synovial membrane and the joint capsule [49]. The earliest known occurrences of spondyloarthropathy in the fossil record date back to the Early Permian and affect the sphenacodontid synapsids *Dimetrodon* and *Ctenorhachis* as well as the stem-amniote *Diadectes* [53]. The latter taxon shows that spondyloarthropathy must have been evolved within tetrapods already in the stem-forms of amniotes. Cisneros et al. [54] described three fused caudal vertebrae in an Early Triassic basal archosaur from South Africa and diagnosed spondarthritis (a frequently used synonym for spondyloarthropathy). According to Rothschild et al. [53], however, the described pathology in this specimen could also be caused by infection. Thus, the presence of spondyloarthropathy in the phytosaur from Halberstadt is the first unambiguous evidence of this disease in a Triassic tetrapod and in a phytosaur. While much of spondyloarthropathy in extant lizards and crocodiles is caused by infectious agent diarrhea such as *Salmonella*, *Shigella*, *Yersinia*, *Campylobacter*, or *Escherichia* [52], the phylogenetic extent of spondyloarthropathy and its apparent increase through geologic time might indicate that this disease is connected with a factor that gives a hidden, so far unknown benefit to its host [25].

## References

[1] Hungerbühler A (2002) The Late Triassic phytosaur *Mystriosuchus westphali*, with a revision of the genus. Palaeontology 45: 377–418.

[2] Stocker MR (2012) A new phytosaur (Archosauriformes, Phytosauria) from the Lot’s Wife beds (Sonsela Member) within the Chinle Formation (Upper Triassic) of Petrified Forest National Park, Arizona. J Vert Paleontol 32: 573–586.

[3] McGregor JH (1906) The Phytosauria, with especial reference to *Mystriosuchus* and *Rhytidodon* . Mem Am Mus Nat Hist 9: 27–100.

[4] Camp CL (1930) A study of the phytosaurs with description of new material from western North America. Mem Univ Calif 10: 1–174.

[5] Case EC (1932) A perfectly preserved segment of the armor of a phytosaur, with associated vertebrae. Contrib Mus Paleontol Univ Mich 4 (2): 57–80.

[6] Gregory JT (1962) The relationships of the American phytosaur *Rutiodon* . Amer Mus Novit 2095: 1–22.

[7] Gregory JT (1969) Evolution und interkontinentale Beziehungen der Phytosauria (Reptilia). Paläontol Z 43: 37–51.

[8] Westphal F (1976) Phytosauria. In: Kuhn O, editor. Handbuch der Paläoherpetologie, vol. 13. Stuttgart: Gustav Fischer Verlag. 99–120.

[9] Chatterjee S (1978) A primitive parasuchid (phytosaur) reptile from the Upper Triassic Maleri Formation of India. Palaeontology 21: 83–127.

[10] Gozzi E, Renesto S (2003) A complete specimen of *Mystriosuchus* (Reptilia, Phytosauria) from the Norian (Late Triassic) of Lombardy (Northern Italy). Riv Ital Paleontol S 109: 475–498.

[11] Stocker MR, Butler RJ (2013) Phytosauria. In Nesbitt SJ, Desojo JB, Irmis RB, editors. Anatomy, phylogeny and palaeobiology of early archosaurs and their kin. Geol Soc London Spec Pub 379: 91–117.

[12] Hunt AP (1989) Cranial morphology and ecology among phytosaurs. In: Lucas SG, Hunt AP, editors. Dawn of the age of dinosaurs in the American Southwest. Albuquerque: New Mexico Museum of Natural History. 349–354.

[13] Sereno PC (1991) Basal archosaurs: phylogenetic relationship and functional implications. Soc Vert Paleontol Mem 2: 1–45.

[14] Brusatte SL, Benton MJ, Desojo JB, Langer MC (2010) The higher-level phylogeny of Archosauria (Tetrapoda: Diapsida). J Syst Palaeontol 8: 3–47.

[15] Benton MJ (2004) Origin and relationships of Dinosauria. In: Weishampel DB, Dodson P, Osmólska H, editors. Dinosauria. Second edition. Berkeley: University of California Press. 7–24.

[16] Nesbitt SJ (2011) The early evolution of archosaurs: relationships and the origin of major clades. Bull Am Mus Nat Hist 352: 1–292.

[17] Jaeger GF (1828) Über die fossilen Reptilien, welche in Württemberg aufgefunden worden sind. Stuttgart: Metzler. 48 p.

[18] Huene F von (1911) Beiträge zur Kenntnis und Beurteilung der Parasuchier. Geol Paläont Abh, NF 10: 67–121.

[19] Moodie RL (1917) Studies in paleopathology. I. General consideration of the evidence of pathological conditions found among fossil animals. Ann Med Hist 1917: 374–381. PMC7927727 33943144

[20] Moodie RL (1918) Paleontological evidences of the antiquity of disease. Scient Month 1918 (9): 265–281.

[21] Moodie RL (1923) The antiquity of disease. Illinois: University of Chicago Press. 148 p.

[22] Abel O (1922) Die Schnauzenverletzungen der Parasuchier und ihre biologische Bedeutung. Paläontol Z 5: 26–57.

[23] Moodie RL (1922) The palaeopathology of the parasuchians. Science 56: 417. 17790198 10.1126/science.56.1450.417

[24] Huene F von (1922) Neue Beiträge zur Kenntnis der Parasuchier. Jb Preuss Geol Landesanst 42: 59–160.

[25] Rothschild BM, Martin LR (2006) Skeletal impact of disease. New Mex Mus Nat Hist Sci Bull 33: 1–226.

[26] Jaekel O (1914) Über die Wirbeltierfunde in der Oberen Trias von Halberstadt. Paläontol Z 1: 155–215.

[27] Hurlburt GR, Heckert AB, Farlow JO (2003) Body mass estimates of phytosaurs (Archosauria: Parasuchidae) from the Petrified Forest Formation (Chinle Group: Revueltian) based on skull and limb bone measurements. New Mex Mus Nat Hist Sci Bull 24: 105–113.

[28] Irmis RB (2007) Axial skeleton ontogeny in the Parasuchia (Archosauria: Pseudosuchia) and its implications for ontogenetic determination in archosaurs. J Vert Paleontol 27: 350–361.

[29] Lautenschlager S, Desojo JB (2011) Reassessment of the Middle Triassic rauisuchian archosaurs *Tichinosuchus ferox* and *Stagonosuchus nyassicus* . Paläontol Z 85: 357–381.

[30] Gower DJ (2001) Possible postcranial pneumacity in the last common ancestor of birds and crocodilians: evidence from *Erythrosuchus* and other Mesozoic archosaurs. Naturwissenschaften 88: 119–122. 11402840 10.1007/s001140100206

[31] Butler RJ, Barrett PM, Gower DJ (2012) Reassessment of the evidence for postcranial skeletal pneumaticity in Triassic archosaurs, and the early evolution of the avian respiratory system. PLOS ONE 7(3): e34094 10.1371/journal.pone.0034094 22470520 PMC3314707

[32] Wilson JA (1999) A nomenclature for vertebral laminae in sauropods and other saurischian dinosaurs. J Vert Paleontol 19: 639–653.

[33] Wilson JA, D’Emic MD, Ikejiri T, Moacdieh EM, Whitlock JA (2011) A nomenclature for vertebral fossae in sauropods and other saurischian dinosaurs. PLOS ONE 6(2): e17114 10.1371/journal.pone.0017114 21386963 PMC3046170

[34] Spielmann JA, Lucas SG (2012) Tetrapod fauna of the Upper Triassic Redonda Formation, East-central New Mexico: the characteristic assemblage of the Apachean land-vertebrate faunachron. New Mex Mus Nat Hist Sci Bull 55: 1–112.

[35] Tsuihiji T (2004) The ligament system in the neck of *Rhea americana* and its implication for the bifurcated neural spines of sauropod dinosaurs. J Vert Paleontol 24: 165–172.

[36] Bruggeman RJ, Maier JA, Mohiuddin YS, Powers R, Lo Y, et al (2012) Avian intervertebral disc arises from rostral sclerotome and lacks a nucleus pulposus: implications for evolution of the vertebrate disc. Dev Dyn 241: 675–683. 22354863 10.1002/dvdy.23750 PMC3302952

[37] Dawes B (1930) The development of the vertebral column in mammals, as illustrated by its development in *Mus musculus* . Phil Trans Roy Soc Lond B 218: 115–170.

[38] Rothschild BM, DePalma RA, Burnham DA, Martin LD (In press) Anatomy of a dinosaur – clarification of vertebrae in vertebrate anatomy. Anat Histol Embryol. 10.1111/ahe.12573 32468658

[39] Baumel JJ, Raikow RL (1993) “Arthrologia”. In: Baumel JJ, King AS, Breazile JE, Evans HE, Vanden Berge JC, editors. Handbook of avian anatomy: Nomina Anatomica Avium. Cambridge, Massachusetts: Nuttal Ornithological Club. 133–187.

[40] Salisbury S, Frey E (2001) A biomechanical transformation model for the evolution of semi-spheroidal articulations between adjoining vertebral bodies in crocodilians. In: Grigg GC, Seebacher F, Franklin CE, editors. Crocodilian biology and evolution. Chipping Norton: Surrey Beatty & Sons. 85–134.

[41] Hoffstetter R, Gasc J-P (1969) Vertebrae and ribs of modern reptiles. In: Gans C, Bellairs A d’A, Parsons TS, editors. Biology of the Reptilia, vol.1. London: Academic Press. 201–310.

[42] Winchester L, Bellairs A d’A (1977) Aspects of vertebral development in lizards and snakes. J Zool 181: 495–525.

[43] Rothschild BM (2005) Osseotypes and spondyloarthropathy exposed. Curr Rheumatol Rev 1: 57–63.

[44] Castriota-Scanderbeg A, Dallapiccola B (2005) Abnormal skeletal phenotypes: from simple signs to complex diagnoses. Heidelberg: Springer. 962 p.

[45] Leivseth G, Frobin W, Brinckmann P (2005) Congenital cervical block vertebrae are associated with caudally adjacent discs. Clin Biomech 20: 669–674. 10.1016/j.clinbiomech.2005.04.006 15964113

[46] Vega CS, Maisch MW (2014) Pathologic features in Upper Permian and Middle Triassic dicynodonts (Synapsida, Therapsida). In: Kammerer CF, Angielczyk KD, Fröbisch J, editors. Early evolutionary history of the Synapsida. Vertebrate Paleobiology and Paleoanthropology. Heidelberg: Springer. 151–161. DOI:10.1007/978-94-007-6841-3_9.

[47] Eysel P, Peters KM (1997) Spondylodiszitis. In: Peters KM, Klosterhalfen B, editors. Bakterielle Infektionen der Knochen und Gelenke. Stuttgart: Enke. 52–68.

[48] Adler CP (2005) Knochenkrankheiten. Diagnostik makroskopischer, histologischer und radiologischer Strukturveränderungen des Skeletts. Third edition. Heidelberg: Springer. 623 p.

[49] Rothschild BM (2009) Scientifically rigorous reptile and amphibian osseous pathology: lessons for forensic herpetology from comparative and paleo-pathology. App Herpetol 6: 47–79.

[50] Resnick D, Niwayama G (1988) Diagnosis of bone and joint disorders. Philadelphia: WB Saunders. 5600 p.

[51] Rothschild BM, Helbling II M, Miles C (2002) Spondyloarthropathy in the Jurassic. Lancet 360: 1454. 12433512 10.1016/s0140-6736(02)11471-1

[52] Rothschild BM (2010) Macroscopic recognition of nontraumatic osseous pathology in the postcranial skeletons of crocodilians and lizards. J Herpetol 44: 13–20.

[53] Rothschild BM, Schultze H-P, Pellegrini R (2012) Herpetological osteopathology. Annotated bibliography of amphibians and reptiles. Heidelberg: Springer. 450 p.

[54] Cisneros JC, Gomes Cabral U, de Beer F, Damiani R, Costa Fortier D (2010) Spondarthritis in the Triassic. PLOS ONE 5(10): e13425 10.1371/journal.pone.0013425 20976231 PMC2954804

[55] Huene F von (1913) A new phytosaur from the Palisades near New York. Bull Am Mus Nat Hist 32: 275–282.

[56] Wettstein O von (1937) 2. Ordnung der Klasse Reptilia: Crocodilia. In: Handbuch der Zoologie. Eine Naturgeschichte der Stämme des Tierreiches, 7. Band: Sauropsida. Allgemeines, Reptilia, Aves, 1. Teilband. Berlin, Leipzig: de Gruyter. 236–424.

